# Detection of circovirus and herpesvirus co-infection in suddenly dead common ravens (*Corvus corax*)

**DOI:** 10.1007/s11259-026-11453-4

**Published:** 2026-08-08

**Authors:** Aleksandra Ledwoń, Rafał Sapierzyński, Beata Dolka, Dorota Witkowska, Małgorzata Gbylik-Sikorska, Bartosz Sell

**Affiliations:** 1 Department of Pathology and Veterinary Diagnostics of the Institute of Veterinary Medicine, Warsaw University of Life Sciences, Warsaw, Poland; 2 Toxicological Diagnostics Research Team, Department of Chemical Research of Food and Feed, National Veterinary Research Institute, Puławy, 24-100 Poland

**Keywords:** Common raven, Raven circovirus, Raven herpesvirus, Wildlife poisoning

## Abstract

This article investigates the causes of the sudden death of six common ravens (*Corvus corax*). All six birds were found on the same day in central Poland. The ravens were necropsied, and samples were collected for bacteriological, histopathological, and toxicological examinations. The presence of genetic material from common viral and some bacterial pathogens was examined using molecular methods. The ravens were found to be in good nutritional condition. Two birds had enlarged spleens with petechiae on their surface. Histopathological examination revealed multifocal hemorrhages and inflammatory infiltrates composed of mononuclear cells in the liver, and intranuclear inclusion bodies in hepatocytes. In the kidneys, inflammation, hemorrhages, parenchymal degeneration, and necrosis of the renal tubular epithelium were detected. Mild lymphocytic-plasmacytic inflammation was found in the intestines. Molecular tests were performed for circoviruses, herpesviruses, flaviviruses, adenoviruses, polyomavirus, avian bornavirus, avian paramyxovirus-1, rotavirus, avian reovirus, avian influenza viruses, *Chlamydia* spp., *Salmonella* spp., *Clostridium* spp., botulin toxins (A-E). Only PCR tests for circoviruses and herpesviruses yielded positive results. Toxicological tests were performed for the presence of organophosphate pesticides, rodenticides, coccidiostats, mycotoxins, and fungicides. Low and trace concentrations of brodifacoum (an anticoagulant rodenticide) and pentobarbital (a barbiturate) were detected. The remaining results were negative. There is a strong suspicion that the primary cause of death in the studied birds was raven herpesvirus, described in this case for the first time, with possible involvement of circovirus. However, other factors, including poisoning or other unknown viruses, cannot be ruled out. Although the toxicology profile was broad, it did not include all possible toxins.

## Introduction

The common raven (*Corvus corax*) is one of the largest passerine birds in the world, and the largest in Poland, where it is partially protected. The raven population in Poland was estimated at 26,000–37,000 pairs in 2008–2012 (Inspekcja Ochrony Środowiska 2019), and the population trend is still upward (Inspekcja Ochrony Środowiska 2024). Common ravens are scavengers and active predators; they are omnivorous generalists that exploit a wide variety of food sources in their foraging territory. Mammals tend to be the most important source of food, with small rodents and shrews predominating (Laudet and Selva 2005). However, they also eat eggs, insects, grains, fruit, garbage, and birds (Boarman and Heinrich 1999).

Outbreaks of fatal viral diseases causing sudden declines in many corvids have been reported in the past. In Japan in 2004, mass mortality of birds, including several hundred large-billed crows (*Corvus macrorhynchos*), was reported and was attributed to the H5N1 avian influenza virus. In Iran, a case of H5N8 influenza was reported in hooded crows (*Corvus cornix*) in 2017. West Nile virus also causes significant losses in corvid populations (Yaremych et al. 2004). In recent years, numerous cases and deaths of corvids and other wild birds caused by this virus have been reported in Poland (Niczyporuk et al. 2023). Epizootic mortality in several hundred American crows (*Corvus brachyrhynchos*) caused by orthoreovirus was recorded between 2001 and 2017 in the USA (Forzán et al., 2019). In 2006, numerous deaths were reported in jackdaws (*Corvus monedula*) in Spain due to co-infection with polyomavirus and *Salmonella* spp. (Johne et al. 2006). Circoviruses have been isolated from other corvids (Stewart et al., 2006; Ledwoń et al., 2025), but so far, these have not been cases of sudden death. Circovirus has been found in Australian ravens (*Corvus coronoides*) with plumage lesions similar to those seen in parrots with viral beak and feather disease (Stewart et al., 2006), in domestic pigeons with feather abnormalities (Dolka et al. 2016) and in the Australian magpie (*Gymnorhina tibicen*) (Nath et al. 2023). In Canada, circovirus infection was found in a common raven with an opportunistic fungal infection (Pesant et al. 2024). Circoviruses were also detected in corvids necropsied in our department, including western jackdaws, hooded crows, magpies, and rooks; the cause of death was not directly related to viral infection (Ledwoń et al., 2025).

Herpesviruses have been reported even less frequently in corvids. Studies of oropharyngeal and cloacal swabs conducted in Slovakia revealed herpesvirus infections in 3 of 40 crows examined, while no infections were found in ravens, jackdaws, or magpies. However, relatively few individuals of these species were tested, only 12 (Žlabravec et al. 2022). Pigeon-type herpesvirus PiHV-1 was also detected in the hooded crow (*Corvus cornix*) (Woźniakowski et al. 2013).

The following case describes the sudden death of six ravens that were also infected with circovirus and herpesvirus.

## Materials and methods

### Case description

In January 2024, six ravens (*Corvus corax*), three females and three males, were brought to the Department of Veterinary Pathology and Diagnostics, Faculty of Veterinary Medicine, Warsaw University of Life Sciences to determine the cause of death. All birds had been found five days earlier in a forest in Radzymin, Masovian Voivodeship, Poland. The bird carcasses were found in the same place, close to each other. Poisoning was initially suspected. The night before the ravens were found, the ambient temperature was -5°C, while during the day it was +1°C (AccuWeather 2024). After the carcasses were discovered, they were stored in a cold storage facility.

### Necropsy

All six ravens underwent post-mortem examination during which material was collected from all birds for histopathological and PCR tests, from five birds for toxicological tests, and from two birds for bacteriological tests. Wet-mount preparations of intestinal contents were prepared from all birds.

### Cytology and bacteriology

 During necropsy, cytological slides of the liver, spleen, lungs, and intestines were prepared from each raven and stained using the Hemacolor® (Merck KGaA, Darmstadt, Germany) and Ziehl-Neelsen (Pol-Aura, Morąg, Poland) methods. Because very small amounts of bacteria were found in the slides, bacteriological testing for aerobic bacteria (Columbia Agar with blood and MacConkey Agar (Graso Biotech, Starogard Gdański, Poland)/48 h/37°C) was performed only on two ravens: 1 and 2.

### Histopathological examination

Samples of the liver, brain, kidneys, spleen, pancreas, and intestines were collected for histopathological examination. Tissue samples were fixed in 10% neutral-buffered formalin, dehydrated in increasing gradients of ethyl alcohol, and embedded in paraffin. Each tissue sample was then cut in the microtome at 4 μm thickness. Finally, paraffin sections were stained with hematoxylin and eosin (H-E). Histopathological analyses were conducted using an Olympus BX41 microscope (Olympus, Tokyo, Japan).

### Toxicology

 For toxicological testing, two pooled samples were collected from five birds: one from the gizzard contents and one from the liver. Toxicological diagnosis was performed using chromatographic methods with mass spectrometry. The study included the presence of toxins such as: organophosphate pesticides (azinphos-ethyl, azinfos-methyl, chlorfenvinfos, chlorpyrifos, diazinon, dichlofenthion, dichlorvos, dicrotophos, dimefox, dimethoate, disulfoton, ethion, etrimfos, foxim, fonophos, formotion, fosalon, malaoxon, malathion, methamidofos, mevinphos, omethoate, dioxacarb, carbofuran, methiocarb, pirimicarb, propoxur), rodenticides (bromadiolone, brodifacoum, chloralose, chlorphacinone, difacinone, difenacum, difethialone, flocoumafen, coumatetraryl, kumachlor, strychnine, warfarin), coccidiostats (lazalocide, maduramycin, monensin, narasin, salinomycin, semdumycin), mycotoxins (aflatoxins B1, B2, G1, G2, deoxynivalenol, ochratoxin A, toxin T2, HT2 toxin, zearalenone) and fungicides. The analyses were performed according to a previously published method by Sell et al. (2018).

### Molecular tests

#### DNA and RNA extraction

Two pooled samples weighing approximately 20 mg were collected from each bird, consisting of sections of brain, liver, spleen, lung, and kidney, and nucleic acid extraction was performed. DNA was isolated using the Genomic Mini AX Tissue Spin kit (A&A Biotechnology, Gdańsk, Poland), and RNA using the Total RNA Mini Plus kit (A&A Biotechnology, Gdańsk, Poland), according to the manufacturer’s instructions. In addition, separate samples of liver, spleen, kidney, brain, lung, and intestinal tissues from raven 1 (for herpesvirus) and liver and intestines from other ravens were individually processed for DNA extraction (for Clostridium and botulin toxins A-E).

#### PCR, RT-PCR and RT-qPCR tests 

 PCR tests were performed using PCR Mix Plus Green (A&A Biotechnology, Gdańsk, Poland). RT-PCR was performed using OneStep RT-PCR (Eurx, Gdańsk, Poland) and RT-qPCR with Brilliant II QRT-PCR MasterMix kit, 1-step (Agilent Technologies, CA, Santa Clara, USA). The tested pathogens, methods, and results are presented in Table 1. RT-PCR products were purified with the GeneMATRIX Agarose-Out DNA Purification Kit (EURx, Gdańsk, Poland) and sent for Sanger sequencing to Genomed (Warsaw, Poland).

Analysis of genetic sequences of selected circovirus and herpesvirus isolates was performed using FinchTV software v.1.4.0 (Geospiza Inc., 2004-2012) and the Molecular Evolutionary Genetics Analysis version 12 (MEGA 12) (Kumar et al., 2024) software.

## Results

### Necropsy

**Fig. 1:**
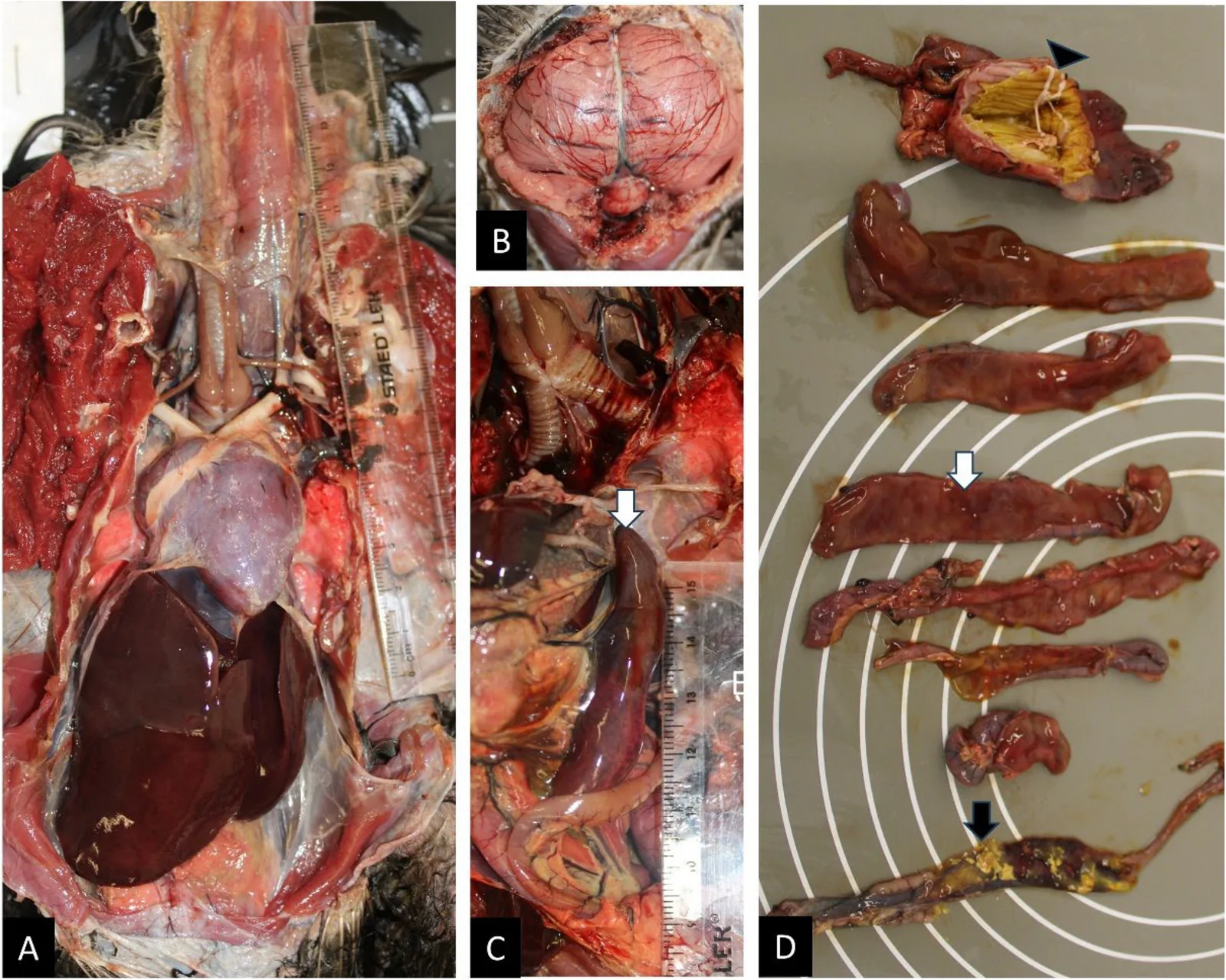
Necropsy of Raven 6: (**A**) Slightly mottled liver with small white foci of necrosis in the right lower third of the lobe. (**B**) Moderately congested brain with dilated meningeal vessels. (**C**) Moderately enlarged spleen (white arrow) with petechiae throughout. (**D**) Small amounts of fibrous material in the gizzard (black arrowhead); scant bloody material in the jejunum (white arrow); yellow urates in the ileum (black arrow)

The following findings were observed in the necropsied ravens: all had moderate “stress lines” on their remiges and rectrices, good pectoral muscle condition, and body weights ranging from 1045 to 1314 g. In females, despite their lower body weight compared to males, more fat was found in the body cavity. All birds had dilated meningeal vessels (Fig. 1B), and the brains were slightly congested. In most birds, the liver was of normal size and consistency. In raven 1, a lighter 1.2 × 0.5 cm lesion was observed on the organ’s surface. In raven 5, the cranial margin of the right lobe was beige, while the caudal margin of the right lobe was rounded, with small grey foci on the organ’s surface. In raven 6, the liver was slightly mottled with two little white foci of necrosis in the right lower third of the lobe (Fig. 1A). In raven 4, a rupture of the right liver lobe with limited hemorrhage (clotting blood) was observed. This bird also had bruising of the pectoral muscles, suggesting trauma, possibly a fall from a height. The gallbladders of five birds were filled with bile. Splenic enlargement and petechiae on its surface were observed in ravens 3 and 6 (Fig. 1C). The kidneys of all birds examined were moderately enlarged. Congestion of varying degrees was observed in the lungs, with the greatest severity observed in raven 4. In all tested ravens, the esophagus and proventriculus were empty. Small amounts of white or creamy-white sinewy mass were usually found in the gizzards of all birds (Fig. 1D). In all ravens examined, the intestines contained a small amount of beige mucous material with a small amount of bloody content, although ravens 3 and 6 (Fig. 1D) had more bloody content. In the terminal section of the ileum, rectum, and cloaca of raven 6, the presence of yellow urates was found (Fig. 1D). The heart ventricles were in full or partial contraction, and the blood in the heart and veins was clotted. The gonads were inactive in all birds. A wet mount examination of the intestinal contents of crow 4 revealed the presence of a few roundworms several millimeters long. In the remaining birds, no parasites were found in the gastrointestinal tract.

### Histopathological examination

**Fig. 2:**
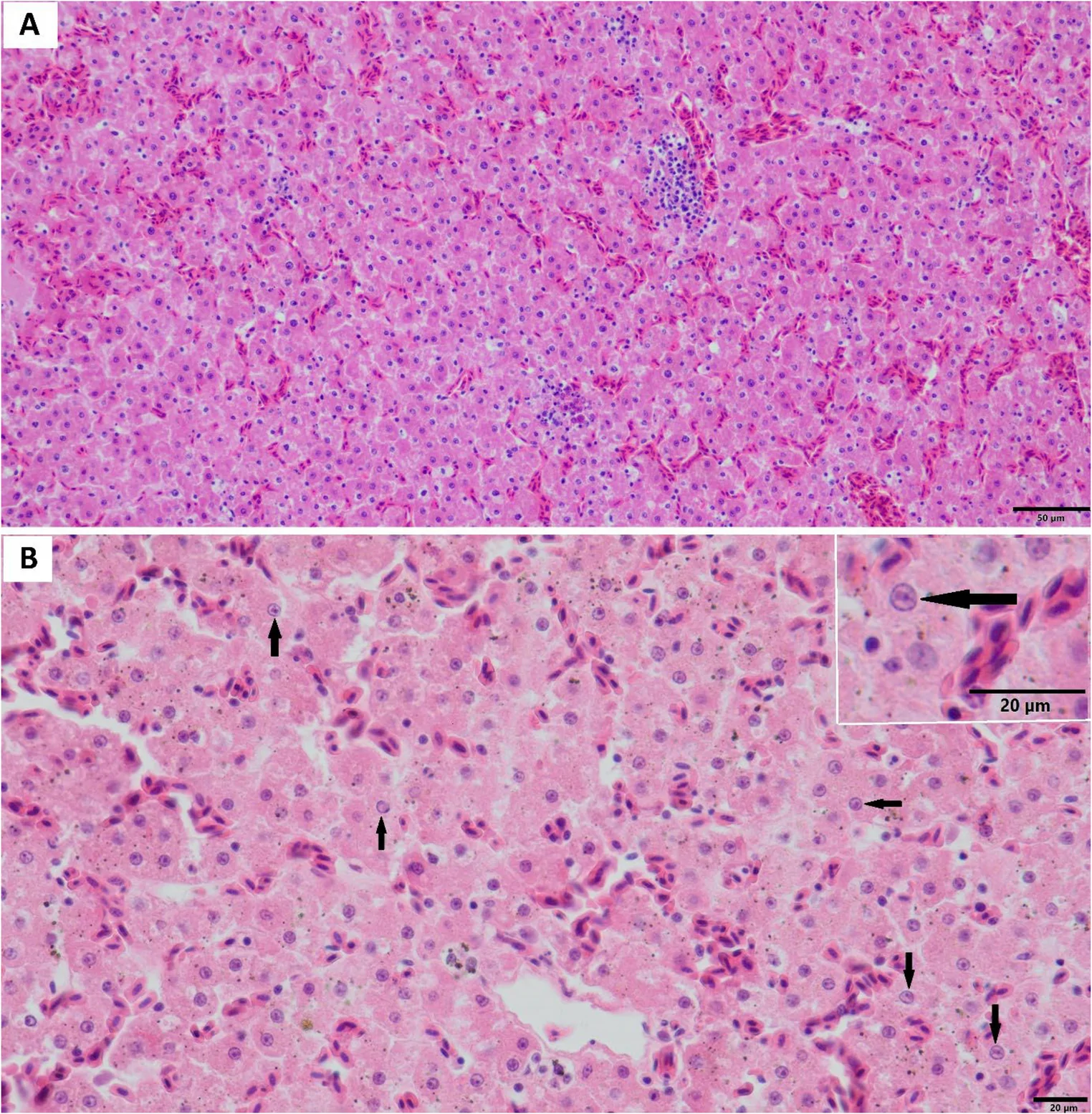
Histopathologic changes in the liver samples collected from raven 1. Figure 2A – hyperemia and multifocal inflammatory infiltrate randomly distributed within liver parenchyma are visible (hematoxylin-eosin, magnification 100x, bar 50 μm). Figure 2B - intranuclear eosinophilic structures resembling intranuclear inclusion bodies (black arrows) are visible in a few hepatocytes; hematoxylin-eosin, magnification 200x (bar 20 μm)

**Fig. 3:**
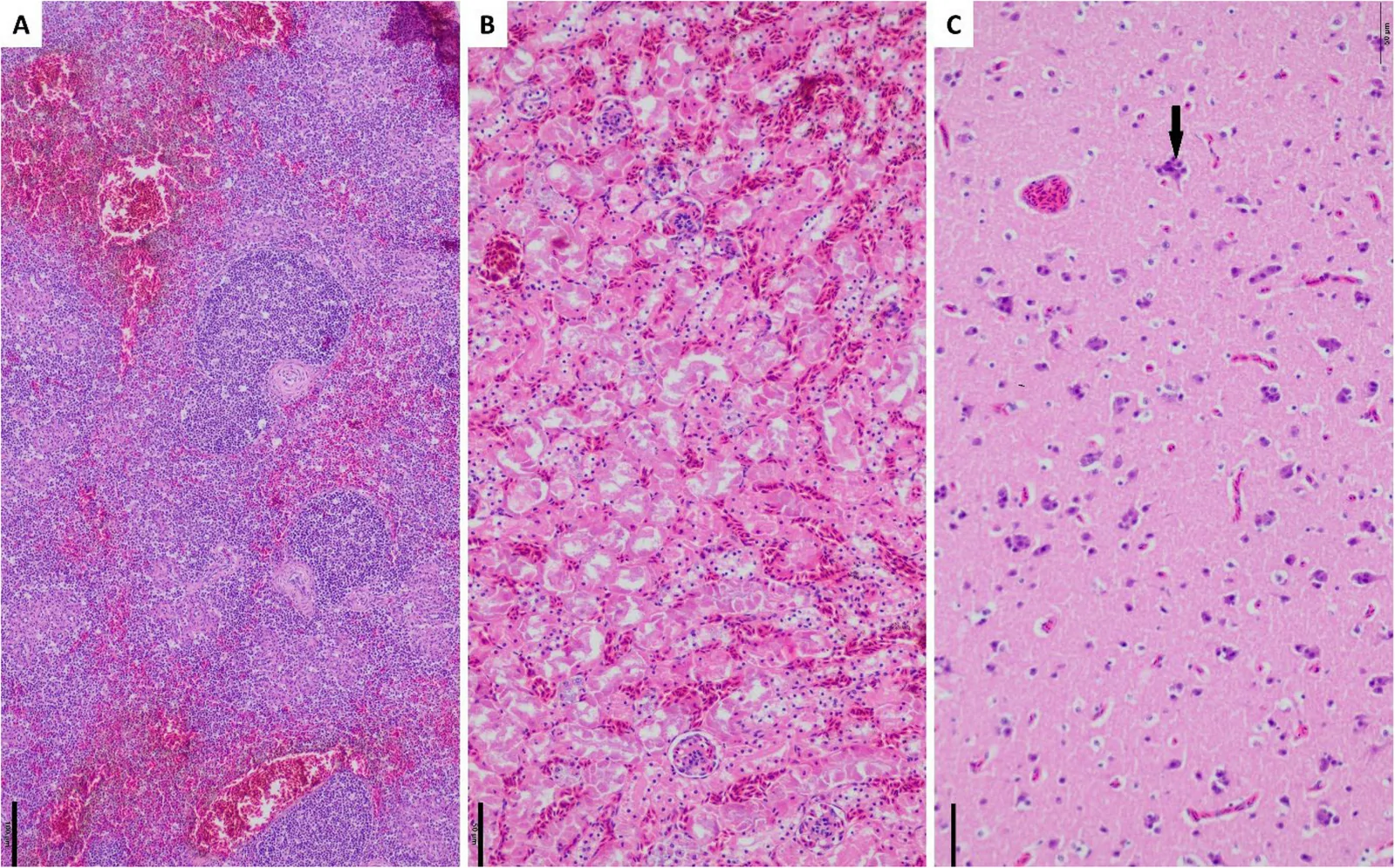
Histopathologic changes in the samples of spleen, kidney and brain collected from ravens 1 and 3. Figure 3A - spleen of raven 3 – hyperemia, hemorrhages, and reactive hyperplasia of splenic lymphoid follicles are visible; hematoxylin-eosin, magnification 40x, (bar 100 μm). Figure 3B - kidney of raven 1 - hyperemia, parenchymal degeneration, and necrosis of renal tubular epithelial cells are visible; hematoxylin-eosin, magnification 100x, (bar 50 μm). Figure 3C - brain of raven 3 - hyperemia, neuronophagy with formation of glial noduli (arrow); hematoxylin-eosin, magnification 100x, (bar 50 μm)

 In four of the six ravens examined, the liver showed blood stasis, multifocal hemorrhages, and multifocal inflammatory cell infiltrates composed of mononuclear cells (Fig. 2A). Furthermore, in these individuals, intranuclear eosinophilic structures resembling intranuclear inclusion bodies were observed in a some hepatocytes (ig 2B). In two ravens, mild diffuse hepatocyte necrosis was observed, with a reactive mixed inflammatory infiltrate. Three of the four spleen samples examined showed congestion, hemorrhages, and reactive hyperplasia of splenic lymphoid follicles (Fig. 3A). In the kidneys, all five samples examined showed congestion, parenchymal degeneration, and necrosis of renal tubular epithelial cells (Fig. 3B). Furthermore, two birds showed multifocal inflammatory cell infiltrates composed of mononuclear cells. One bird showed only congestion. The intestinal lesion observed in the small intestines in three of the five samples examined was mild lymphocytic-plasmacytic inflammation. In five of the six ravens, brain hyperemia, microglial hyperplasia, and moderate neuronophagia were observed, with the formation of glial nodules (Fig. 3C).

### Cytology and bacteriology. 

Pulmonary filariasis was observed in two birds: 2 and 4. No bacteria were found in the assessed organs, and only a few in the intestines. Ziehl-Neelsen stained slides were negative. Bacteriological examination revealed no bacterial growth.

### Toxicology

The study revealed trace amounts of pentobarbital < 125 µg/kg and brodifacoum 68.7 µg/kg in the livers; these substances were not detected in the stomach contents.

### Molecular tests of organ samples taken from the examined ravens

**Fig. 4:**
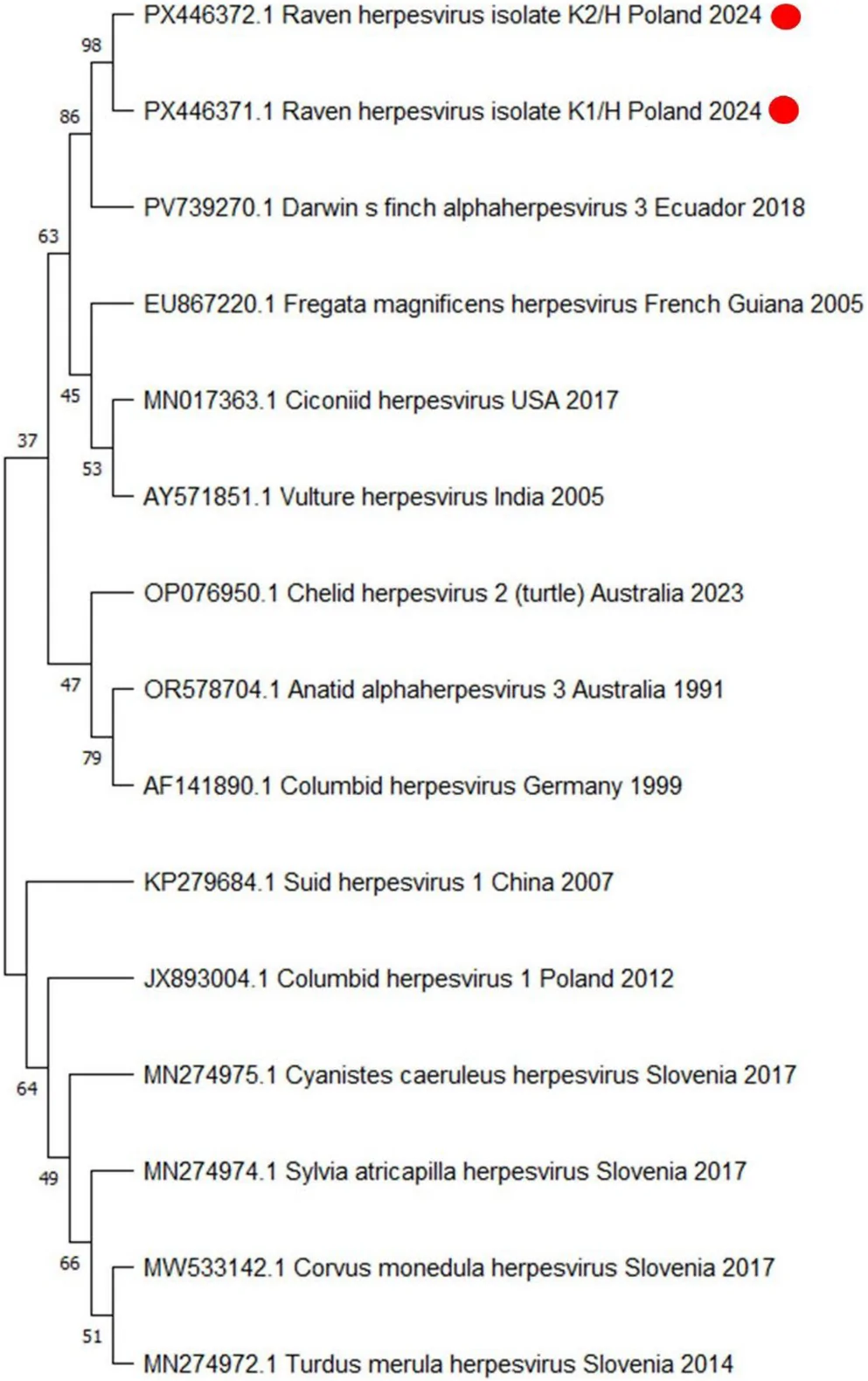
The relationships of raven herpesvirus strains detected in this study investigation (red dots) and other herpesviruses which sequences have been derived from GenBank database are based on the herpesviral DNA polymerase gene nucleotide sequences. The evolutionary history was inferred using the Neighbor-Joining method. The bootstrap consensus tree inferred from 1000 replicates is taken to represent the evolutionary history of the taxa analyzed. Branches corresponding to partitions reproduced in less than 50% bootstrap replicates are collapsed. The percentage of replicate trees in which the associated taxa clustered together in the bootstrap test (1000 replicates) is shown next to the branches. The evolutionary distances were computed using the Maximum Composite Likelihood method and are in the units of the number of base substitutions per site. This analysis involved 15 nucleotide sequences. Codon positions included were 1st+2nd+3rd+Noncoding. All ambiguous positions were removed for each sequence pair (pairwise deletion option). There were a total of 196,405 positions in the final dataset. Evolutionary analyses were conducted in MEGA11 (Tamura et al. 2021)

**Fig. 5:**
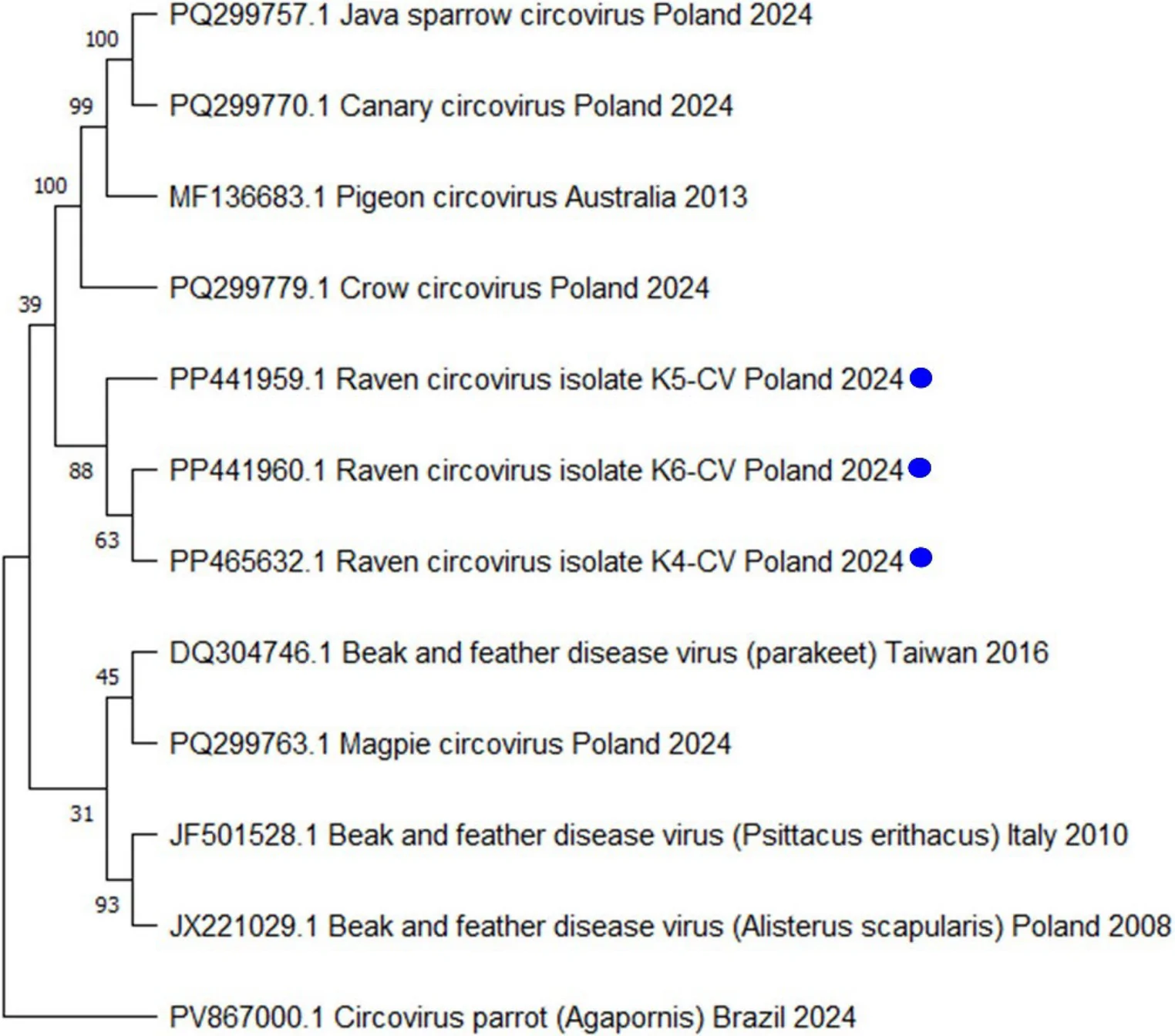
The relationships of raven circovirus strains detected in this study (blue dots) and other circoviruses which sequences have been derived from the GenBank database are based on the Rep-encoding region. The evolutionary history was inferred using the Neighbor-Joining method. The bootstrap consensus tree inferred from 1000 replicates is taken to represent the evolutionary history of the taxa analyzed. Branches corresponding to partitions reproduced in less than 50% bootstrap replicates are collapsed. The percentage of replicate trees in which the associated taxa clustered together in the bootstrap test (1000 replicates) is shown next to the branches. The evolutionary distances were computed using the Maximum Composite Likelihood method and are in the units of the number of base substitutions per site. This analysis involved 12 nucleotide sequences. Codon positions included were 1st+2nd+3rd+Noncoding. All ambiguous positions were removed for each sequence pair (pairwise deletion option). There were a total of 2002 positions in the final dataset. Evolutionary analyses were conducted in MEGA11 (Tamura et al. 2021)

The pooled organ samples yielded positive results for herpesviruses (Fig. 4) and circoviruses (Fig. 5) in all ravens examined (Table 1). In organ samples tested separately from raven 1 for herpesviruses, positive results were obtained from the liver, spleen, and brain, weakly positive results from the lungs and kidneys, and equivocal results from the intestines. All other tests were negative. Three circovirus sequences and two herpesvirus sequences were entered into the GenBank database under the numbers PP465632 (K4), PP441959 (K5), PP441960 (K6) and PX446371 (K1), PX446372 (K2), respectively.

**Table 1:** A list of pathogens tested for presence in tissues of dead ravens using PCR, RT-PCR and RT-qPCR. Positive results were obtained only in tests detecting broad-spectrum circoviruses and herpesviruses

Pathogen tested	Method	Result
circovirus	Nested PCR (Halami et al. 2008)	**+**
herpesvirus	Nested PCR (Van Devanter et al. 1996)	**+**
flavivirus	RT-PCR (Kuno 1998); RT-qPCR (Moureau et al. 2007)	**-**
adenovirus	PCR Raue and Hess 1998	**-**
polyomavirus	Nested PCR (Johne et al. 2005)	**-**
bornavirus	RT-PCR (Kistler et al. 2008)	**-**
avian paramyxovirus-1	RT-PCR (Creelan et al. 2002)	**-**
rotavirus	RT-PCR (Day et al. 2007)	**-**
avian reovirus	RT-PCR (Bruhn et al. 2005)	**-**
influenza A viruses and H5 subtype AIV	RT-qPCR (Spackman et al. 2002); RT-PCR and RT-qPCR (Slomka et al. 2007)	**-**
*Chlamydia* spp.	PCR (Amin et al., 2003)	**-**
*Salmonella* spp.	PCR (Rahn et al., 1992)	**-**
*Clostridium* spp.	PCR (Amit-Romach et al. 2004; Mirhosseini et al. 2010)	**-**
Botulin toxins (A-E)	PCR (Szabo et al. 1993)	**-**

## Discussion and conclusions

The ravens described in this report died suddenly in one location, likely during a night’s rest, and therefore, the initial suspicion was poisoning. However, toxicology testing revealed only traces of toxic substances such as brodifacoum and pentobarbital.

Brodifacoum (3-[3-(40-bromobiphenly-4-yl)-1,2,3,4-tetrahydro-1-naphthyl]-1/4 4hydroxycoumarin) is one of the most frequently used second-generation anticoagulant rodenticides (SGAR) on the world market (Stone et al. 1999; Reding and Arent, 2008) and frequently detected toxic substance that affects the health of wild birds of prey in the United States (Redig and Arent 2008) and Poland (Sell et al. 2022). A single toxic dose of brodifacoum (BRF) will usually result in rodent death in 4 to 5 days. Animals can carry sublethal levels of BRF. In the case of wild raptors, exposure may predispose them to predation, traumatic injuries, and reduced hunting ability. Thus, mortality can occur indirectly from exposure to these toxic agents, and therefore, the full impact of rodenticide use for raptors is not realized (Redig and Arent 2008). In toxicological studies conducted in Poland in suspected corvids between 2018 and 2022, BRF and/or bromadiolone (BRL) were detected in the liver of 18 of 22 ravens and 3 of 7 other corvids. BRF concentrations ranged from 27.8 to 453.2 µg/kg, with 4 cases exceeding 100 µg/kg, and BRL concentrations ranged from 5.4 to 70 µg/kg (Sell 2024). However, such studies have been performed in birds of prey Wand have shown the presence of SGAR in up to 74% of birds that died from various causes (Thomas et al. 2011). In Poland a full prevalence (100%) of AR was observed in white-tailed eagles, while half of the birds had liver concentrations exceeding 100 µg/kg (Sell et al. 2022). In barn owls diagnosed postmortem as having died from rodenticides, liver concentrations > 0.1 mg/kg were found, and 0.2 mg/kg already carries a significant risk of acute poisoning (Thomas et al. 2011). In the case of the ravens we studied, this figure was 0.0687 mg/kg. Therefore, the fact that the cause of death in the ravens studied was not rodenticide poisoning is evidenced not only by the lack of typical post-mortem and histopathological signs, but also by the low BRF concentration in the livers. Barbiturate poisoning in scavenging birds is one of the more common cases and results from improper disposal or handling of carcasses of animals after euthanasia (Herrero-Villar et al. 2021).

Median pentobarbital concentrations detected in intoxicated griffon vultures (*Gyps fulvus*) were 38.1 mg/g in the liver (Herrero-Villar et al. 2021), while in the ravens we examined, the concentration in the liver was < 0.000125 mg/g, so death was not caused by barbiturate poisoning.

After ruling out a bacterial infection and considering a toxic agent unlikely, a viral infection was suspected. Among the viruses tested, additional results were obtained in PCR tests for circoviruses and herpesviruses. In the circovirus-positive raven described by Pesant et al. (2024), which also had respiratory mycosis and cachexia, bursal lymphoid depletion with inclusion bodies. It is well known that circoviruses cause lymphocyte depletion with subsequent immunosuppression in several species of mammals and birds (Fehér et al. 2023). The ravens we examined were already adults. Therefore, it was no longer possible to examine the bursa of Fabricius. However, no lymphocyte depletion in the spleen was observed, which is not typical for circovirus disease (Feher et al., 2023). The “stress lines” visible on the flight feathers and rectrices, relatively often observed by us in corvids, could be the result of methionine deficiency during the moulting period (McDonald 2006) or stress experienced at that time; some authors suggest that circovirus infection may also contribute to their formation (Schmidt and Lightfoot 2006). The second virus detected by PCR was a herpesvirus. Herpesviruses have not been described in ravens, but are known to be pathogenic to other birds, such as falcons (Greenwood and Cooper 1982), owls (Phalen et al. 2011; Rose et al. 2012), parrots, and poultry (Forbes and Simpson, 1997). The herpesvirus causing mortality in raptors was found to be closely related to the pigeon herpesvirus (Phalen et al. 2011; Rose et al. 2012). The PCR product obtained was not similar to the pigeon virus, but using discontiguous megablast, some similarity (percent identity: 74.36%) was found to stork herpesviruses (*Ciconia ciconia*) (Mack et al. 2020), Darvin’s Finch alphaherpesvirus 3 (Nieto-Claudin et al., 2026 ), sulid herpesvirus 1 (Niemeyer et al. 2017), anatid alphaherpesvirus-3 (Kelly-Bosma et al. 2024), *Asio otus* and *Strix uralensis* herpesviruses (Žlabravec et al. 2022), vultures (Cardoso et al. 2005), and pigs (Yuan et al. 2025). Of these, most birds did not show any symptoms of the disease, while the disease was found in a young stork (*Ciconia ciconia*) (Mack et al. 2020) and wild Anatidae (Kelly-Bosma et al. 2024). In the case described by Mack et al. (2020), the stork showed progressive weight loss lasting 3 weeks, ending in death. The most characteristic histopathological findings in the stork mentioned were brain lesions involving the formation of glial nodules, necrosis of the spleen and liver, and the presence of intranuclear inclusion bodies in hepatocytes and spleen cells (Mack et al. 2020). In the ravens we studied, death occurred suddenly, and the birds were adults. Therefore, the clinical picture, in terms of the lack of age predilection and its rapid course, was more reminiscent of herpesvirus in Anseriformes. The virus we detected showed some similarity to the Anatidae virus isolated from the black swan (AnHV-3). Cases of herpesvirus in adult owls comparable to those we observed have been described in Australia. Gross lesions in the owls included a pale, normal-sized to enlarged liver, mottling and enlargement of the spleen, and hemorrhagic mucosa of the duodenum, caeca, and colon (Phalen et al. 2011). In the ravens we examined, the liver was most often of normal size, mottling and enlargement of the spleen were observed in two ravens, and hemorrhagic lesions in the intestines were more advanced in two individuals.

In summary, there is a strong indication that the main cause of death in the studied birds was viral coinfection. It should also be added that the low temperatures in January may have contributed to the premature death of the disease-weakened ravens. However, other factors, including poisoning, cannot be ruled out. While the toxicological profile was broad, it did not include all possible toxins. Additionally, subclinical concentrations of anticoagulant rodenticides may increase the permeability of blood vessels, which may facilitate the penetration of infectious agents. Similarly, viruses were examined; those that could cause sudden death in corvids were not fully identified. Nevertheless, this is the first time that raven herpesvirus has been detected in this case.

## Data Availability

No datasets were generated or analysed during the current study.
