# Correction: Detection of circovirus and herpesvirus co-infection in suddenly dead common ravens (*Corvus corax*)

**DOI:** 10.1007/s11259-026-11466-z

**Published:** 2026-08-19

**Authors:** Aleksandra Ledwoń, Rafał Sapierzyński, Beata Dolka, Dorota Witkowska, Małgorzata Gbylik-Sikorska, Bartosz Sell

**Affiliations:** 1 Department of Pathology and Veterinary Diagnostics of the Institute of Veterinary Medicine, Warsaw University of Life Sciences, Warsaw, Poland; 2 Toxicological Diagnostics Research Team, Department of Chemical Research of Food and Feed, National Veterinary Research Institute, Puławy, 24-100 Poland

**Correction to: Veterinary Research Communications (2026) 50:512**

**https://doi.org/10.1007/s11259-026-11453-4**

In this article, the title was incorrectly given as 'Detection of circovirus and herpesvirus co-infection in suddenly dead' but should have been 'Detection of circovirus and herpesvirus co-infection in suddenly dead common ravens (*Corvus corax*)'.

The original article has been corrected.

